# Does Side of Onset Influence the Pattern of Cerebral Atrophy in Parkinson’s Disease?

**DOI:** 10.3389/fneur.2016.00145

**Published:** 2016-09-12

**Authors:** Maria C. A. Santos, Lidiane S. Campos, Rachel P. Guimarães, Camila C. Piccinin, Paula C. Azevedo, Luiza G. Piovesana, Brunno Machado De Campos, Augusto C. Scarparo Amato-Filho, Fernando Cendes, Anelyssa D’Abreu

**Affiliations:** ^1^Neuroimaging Laboratory, Department of Neurology, State University of Campinas – UNICAMP, Campinas, São Paulo, Brazil; ^2^Department of Radiology, State University of Campinas – UNICAMP, Campinas, São Paulo, Brazil

**Keywords:** Parkinson’s disease, gray matter atrophy, laterality, voxel-based morphometry, neuroimaging

## Abstract

**Background:**

Imaging studies have revealed widespread neurodegeneration in Parkinson’s disease (PD), but only a few considered the issue of asymmetrical clinical presentations.

**Objective:**

To investigate if the side of onset influences the pattern of gray matter (GM) atrophy in PD.

**Methods:**

Sixty patients (57.87 ± 10.27 years) diagnosed with idiopathic PD according to the U.K. Brain Bank criteria, 26 with right-sided disease onset (RDO) and 34 with left-sided disease onset (LDO), were compared to 80 healthy controls (HC) (57.1 ± 9.47 years). We acquired T1-weighted images on a 3 T scanner. Images were processed and analyzed with VBM8 (SPM8/Dartel) on Matlab R2012b platform. Statistic assessments included a two-sample test (family-wise error *p* < 0.05) with extent threshold of 20 voxels.

**Results:**

Compared to HC, LDO patients had GM atrophy in the insula, putamen, anterior cingulate, frontotemporal cortex, and right caudate, while the RDO group showed atrophy at the anterior cingulate, insula, frontotemporal, and occipital cortex.

**Conclusion:**

This study revealed widespread GM atrophy in PD, predominantly in the left hemisphere, regardless of the side of onset. Future investigations should also consider handedness and side of onset to better characterize cerebral involvement and its progression in PD.

## Introduction

Parkinson’s disease (PD) is well known for its asymmetrical presentation of motor signs, such as tremor, rigidity, and bradykinesia (1). However, the association between PD side of onset, clinical characteristics, and brain morphological alterations are not clear.

Studies have suggested that the side of motor onset is associated with asymmetric brain changes in early PD, worse in the contralateral side (2, 3). Functional imaging showed a reduced cerebral lateralization pattern in PD (4). Patients with left-sided disease onset (LDO) present thinning of motor-related areas in the contralateral hemisphere (5), while patients with predominantly right-sided disease onset (RDO) had a lower dopamine transporter uptake in the left putamen compared to the right putamen, suggesting that asymmetry of disease is not random (6).

However, there is no consensus if cerebral asymmetrical pathology is related to clinical symptoms or signs (7–10). No definitive pattern of differential brain involvement has been reported in asymmetric disease. Our goal was to perform an exploratory analysis to investigate the relationship between cortical gray matter (GM) atrophy and motor asymmetry in PD.

## Materials and Methods

### Participants

The Ethics Committee of the University of Campinas (UNICAMP) Hospital approved the study, and all participants signed an informed consent before any study related procedure. Sixty patients (57.87 ± 10.27 years) with PD, diagnosed according to the UK Parkinson’s Disease Society Brain Bank criteria were consecutively recruited at the Movement Disorders Outpatient Clinic, a major tertiary hospital with a catchment area of six million subjects. No subjects declined participation in the study. All underwent a complete clinical evaluation, including family history, review of PD history, physical and neurological examination, Unified Parkinson’s Disease Rating Scale (UPDRS), Modified Hoehn and Yahr (HY), Scales for Outcomes of Parkinson’s disease (SCOPA), Non-motor symptom scale (NMSS), and Schwab and England Activities of Daily Living, performed by Movement Disorders Specialists. No patient was present with significant language deficit. Patients with bilateral onset or axial symptoms were excluded. Forty-five PD subjects declared right-hand dominance (88.24%), six declared left-hand dominance, and the information was not available for nine.

Side of onset was defined by the patient and family interview as the side that was first symptomatic. This information was confirmed in the medical charts. Most patients in our clinic are under follow up since diagnosis, and this information is highly reliable. We did not take into consideration the asymmetry at the time of evaluation. Twenty-six patients reported RDO, and 34 reported LDO. Concordance between side of onset and self-declared hand dominance was 52.94% (Pearson chi-square = 2.22, *p* = 0.136).

The control group included 80 healthy controls (HC) (57.1 ± 9.47 years) with no history of neurologic or psychiatric disorders and a normal neurologic examination, and they came from the same base population (Campinas metropolitan area, SP-Brazil). There was no evidence of mild cognitive impairment or dementia using DSM V criteria in this group. Hand dominance information was not available for these subjects. We compared the clinical characteristics between patients and controls using a multivariate model, controlling for age, sex, and disease duration, and we did not observe any significant statistical difference (Table 1).

**Table 1:** **Demographic and clinical data of PD patients and HC**.

Groups	RDO	LDO	Total	*p* value	HC
*N*	24	36	60	–	80
Age (years)	56.77 ± 11.42	58.71 ± 9.38	57.87 ± 10.27	0.474	57.1 ± 9.47
Sex (male)	19	24	43	–	31
DD (years)	6.61 ± 7.93	8.68 ± 5.50	7.78 ± 6.68	0.185	
HY	2.31 ± 1.15	2.66 ± 0.85	2.51 ± 0.99	0.107	
UPDRS	33.73 ± 19.67	36.82 ± 17.63	35.48 ± 18.44	0.524	
SCHWAB (%)	73.46 ± 23.99	72.06 ± 19.97	72.67 ± 21.62	0.806	
SCOPA	19.92 ± 5.97	19.59 ± 6.11	20.09 ± 5.43	0.836	
NMSS	68.31 ± 50.35	64.18 ± 41.92	65.97 ± 45.40	0.730	

*N, absolute number of subjects per group; DD, disease duration; HY, Hoehn and Yahr; UPDRS, Unified Parkinson’s Disease Rating Scale; SCHWAB, Schwab and England Activities of Daily Living; SCOPA, Scales for Outcomes of Parkinson’s disease; NMSS, Non-motor symptom scale; RDO, right-sided disease onset; LDO, left-sided disease onset; HC, healthy control*.

For the statistical analysis, we used STATA 13.1 version. Level of significance was established at *p* < 0.05.

### Image Acquisition and Analysis

We acquired anatomical T1-weighted MRI images with isotropic voxels of 1 mm in the sagittal plane on a 3 T Achieva MR unit-PHILIPS Intera^®^ scanner, release 2.6.1.0. The imaging protocol included the following parameters: 1 mm thick, flip angle 8°, TR 7.1, TE 3.2, matrix 240 × 240, and FOV 240 mm × 240 mm. A neuroradiologist analyzed the images in a blinded fashion, to identify movement artifacts and pathological abnormalities, which would lead to exclusion of the subjects from the study. None were excluded at this point.

We converted images from Dicom to the Nifti format using DCM2Nii.^1^ We placed the center point on the anterior commissure and aligned the images using the display button, available on SPM 8 (11).

We used VBM8 toolbox of the statistical parametric mapping (SPM8)^2^ and the Diffeomorphic Anatomical Registration Exponentiated Lie Algebra (Dartel) software on Matlab R2012b platform to process and analyze the images. VBM allows for a voxel-wise comparison of local GM differences between two groups (12). Briefly, VBM-optimized procedure (11) involves, first, the segmentation of the original structural MRI image in native space in GM, white matter (WM), and cerebrospinal fluid (CSF) tissues. Next, the software normalizes GM and WM images to templates in stereotactic space to acquire optimized normalization parameters, which are applied to the raw images. Then, we performed an automatic segmentation of the normalized images. GM images were smoothed using an 8-mm full width at half maximum (FWHM) isotropic Gaussian kernel. Finally, we performed a statistical analysis, employing the general linear model (GLM). We obtained the results on statistical parametric map showing regions of GM concentration with significant differences between the experimental groups (13). Correction for multiple comparisons used the Theory of Random Fields (12).

We displayed the results using SPM12 on Matlab R2014b and xjView (Human Neuroimaging Lab, Baylor College of Medicine, Houston, TX, USA). We accessed group comparisons by SPM through the family-wise error (FWE) at a threshold of *p* < 0.05, corrected for multiple comparisons, with an extent threshold of *K* = 20 voxels. The brain areas were localized according to the automated anatomical labeling available on The Online Brain Atlas Reconciliation Tool (OBART) (14).

Primary analysis included RDO vs. HC and LDO vs. HC. A homogeneity test using covariance excluded one RDO patient.

## Results

There were no statistical significant differences between RDO and LDO groups in HY, NMSS, and Schwab and England scale scores (Table 1).

### LDO vs. HC

**Figure 1:**
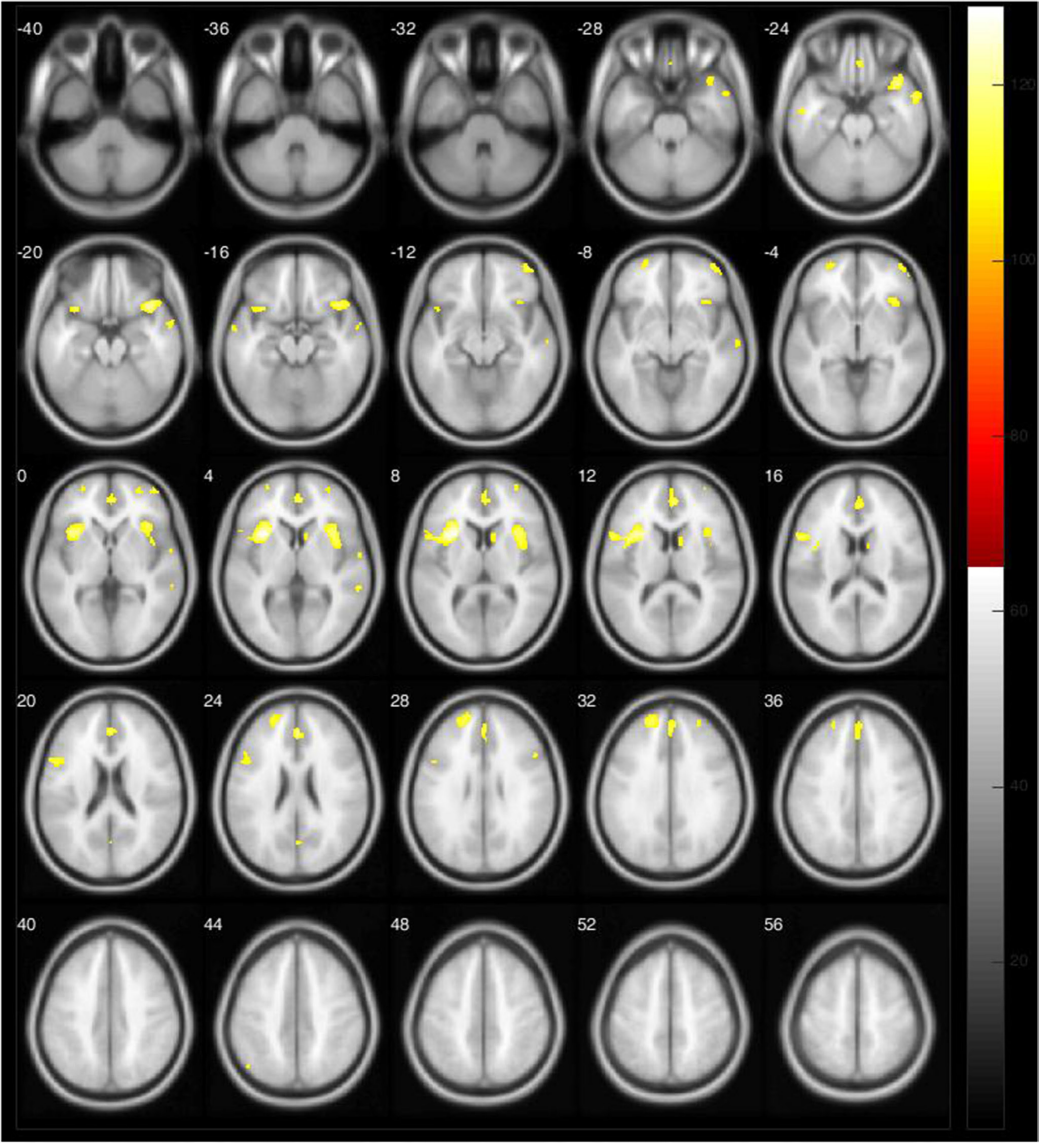
**Areas with GM atrophy labeled in yellow in VBM analysis of LDO PD patients vs. HC**.

Brain atrophy was slightly predominant in the left hemisphere, involving the medial superior temporal pole, frontal lobe, insula, rolandic operculum, putamen, and anterior cingulate. In the right hemisphere, we observed GM atrophy in the medial and superior temporal poles, insula, frontal lobe, putamen, anterior and medium cingulate, and caudate (*p* < 0.05, FWE corrected) (Table 2, Figure 1).

**Table 2:** **Areas with GM atrophy in VBM analyses of RDO vs. HC and LDO vs. HC**.

MNI coordinates (*X*, *Y*, *Z*)	*p* value FWE	*T*-score	*z*-score	Number of voxels	Localization (AAL)
**LDO vs. HC**					
57, 5, −24	0.001	5.76	5.37	228	Temporal_Mid_R (176)
					Temporal_Pole_Mid_R (52)
39, 17, −21	0.000	6.69	6.11	1678	Insula_R (796)
					Temporal_Pole_Sup_R (379)
					Frontal_Inf_Orb_R (229)
					Frontal_Inf_Oper_R (56)
					Putamen_R (33)
					Frontal_Inf_Tri_R (6)
−35, 15, −18	0.001	5.82	5.42	166	Temporal_Pole_Sup_L (60)
					Frontal_Inf_Orb_L (43)
					Insula_L (29)
47, 56, −9	0.001	5.87	5.45	257	Frontal_Mid_Orb_R (212)
					Frontal_Mid_R (29)
					Frontal_Inf_Orb_R (7)
−23, 62, −9	0.003	5.54	5.19	166	Frontal_Sup_Orb_L (92)
					Frontal_Mid_Orb_L (37)
					Frontal_Mid_L (19)
					Frontal_Sup_L (18)
−30, 14, 7	0.000	7.45	6.68	1965	Insula_L (1019)
					Frontal_Inf_Oper_L (588)
					Frontal_Inf_Tri_L (124)
					Rolandic_Oper_L (50)
					Putamen_L (12)
2, 39, 22	0.000	6.42	5.89	1002	Cingulum_Ant_L (307)
					Cingulum_Ant_R (266)
					Frontal_Sup_Medial_L (235)
					Frontal_Sup_Medial_R (160)
					Frontal_Med_Orb_R (18)
					Cingulum_Mid_R (4)
					Frontal_Med_Orb_L (1)
33, 60, 10	0.010	5.25	4.94	104	Frontal_Mid_R (66)
					Frontal_Sup_R (25)
					Frontal_Sup_Orb_R (9)
					Frontal_Mid_Orb_R (4)
9, 11, 9	0.013	5.17	4.88	145	Caudate_R (145)
−20, 48, 31	0.001	5.95	5.52	467	Frontal_Sup_L (267)
					Frontal_Mid_L (200)
**RDO vs. HC**					
−35,−93, −0	0.000	4.04	3.88	42	Occipital_Mid_L (24)
56, −33, −3	0.000	4.09	3.92	39	Temporal_Mid_R (39)
−3, 59, −2	0.000	4.22	4.04	58	Frontal_Med_Orb_L (38)
					Frontal_Sup_Medial_L (20)
−33, 12, 7	0.000	4.49	4.27	199	Insula_L (180)
					Frontal_Inf_Oper_L (13)
42, −6, 10	0.000	4.37	4.17	103	Rolandic_Oper_R (71)
					Insula_R (32)
0, 53, 15	0.000	4.74	4.49	204	Frontal_Sup_Medial_L (173)
					Cingulum_Ant_L (21)
					Cingulum_Ant_R (6)
					Frontal_Sup_Medial_R (2)
17, −96, 28	0.000	4.16	3.98	27	Occipital_Sup_R (21)

*Total of voxels corresponds to the number of voxels of each cluster (*p* < 0.05 FWE)*.

*Height threshold: T = 4.80 and T = 3.86, respectively; Extent threshold: K = 20 voxels; voxel size: 1 mm^3^*.

### RDO vs. HC

**Figure 2:**
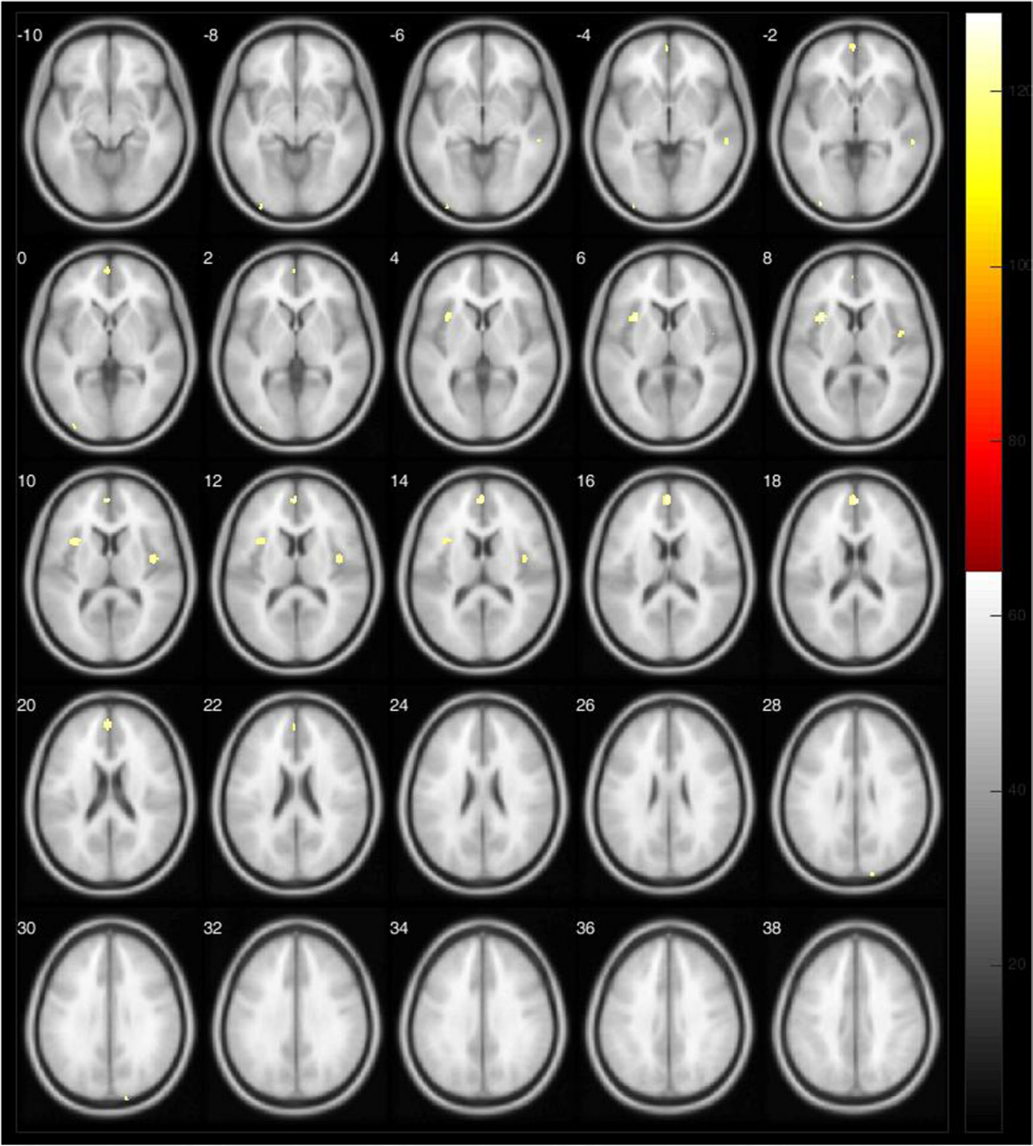
**Areas with GM atrophy labeled in yellow in VBM analysis of RDO PD patients vs. HC**.

Compared with HC, we identified GM atrophy in PD mainly in left hemisphere, including the insula, frontal lobe, and anterior cingulate. In right hemisphere, GM reduction was localized in the temporal medial gyrus, the rolandic operculum, insula, anterior cingulate, and occipital superior gyrus (*p* < 0.05, FWE corrected) (Table 2, Figure 2).

## Discussion

This exploratory study aimed to define GM changes in PD patients, taking into account the asymmetrical onset of the motor signs. Comparing both the LDO to HC and the RDO to HC, the atrophic areas were predominantly localized in the left hemisphere. We observed fewer clusters of GM atrophy in the RDO group than in the LDO group (Table 2, Figure 1). In the LDO group, we identified atrophy in 5773 voxels, 2672 voxels in the right hemisphere (46.28%) and 3101 voxels in the left (53.72%), while in the RDO group, we found atrophy in only 640 voxels, 469 in the left hemisphere (73.28%) and 171 voxels in the right hemisphere (26.72%). We did not observe significant differences between both groups regarding age, disease duration, and disease severity, as measured by clinical scales. The two central findings of this study are (1) brain changes are asymmetric, but not necessarily worse contralateral to the side of onset of the disease and (2) there is greater structural brain atrophy in patients with LDO than RDO.

The motor signs and symptoms of PD are initially unilateral in most cases, corresponding to stage 1 of HY scale. Therefore, an asymmetric pattern of brain damage is an intuitive expectation although motor severity may be more related to basal ganglia rather than cortical changes (2). As disease duration increases, PD tends to become more symmetrical (15).

Our findings corroborate clinical studies suggesting clinical differences between LDO and RDO. The side of disease onset and handedness appear to influence disease progression and its features, including non-motor symptoms (16, 17). LDO may be associated with more significant cognitive decline and reduced goal-directed behavior (18). Left-handed LDO is related to longer disease duration (19), whereas patients with RDO usually have difficulties with language-related tasks and verbal memory. LDO patients with predominant rigidity–bradykinesia have a worse prognosis than RDO with predominant tremor (20–22). Moreover, the left hemisphere appears to have a stronger association with the ipsilateral side of the body than the right one (23).

Our findings also strengthen the hypothesis of asymmetrical neurodegeneration in PD. Asymmetries in the substantia nigra (SN) and the putamen in early PD have been linked to unilateral motor symptoms (2). One analysis showed reduction of dopamine transporter availability in bilateral putamen; however, the decrease was greater in the left putamen in right-handed RDO (6). RDO with mild cognitive impairment had a reduction of the left thalamus volume compared to LDO, which suggests that dominance of motor manifestations might be associated with a pattern of lateralized brain loss (24). LDO patients have worse visual–spatial performance compared to RDO, and GM decrease was observed mainly in the hemisphere contralateral to the side of disease onset, primarily in the right middle frontal gyrus and the precuneus (10). Subjects with right-handed RDO have less cortical thinning than right-handed LDO, suggesting a possible neuroprotective effect of handedness on the contralateral motor cortex (5).

The major limitation of our study was the lack of adjustment for handiness in the analysis. This information was lacking for some subjects, and had we performed the analysis using four subgroups, the results would probably be meaningless due to the small size of each sample. Due to the exploratory nature of this study and since most imaging studies in PD do not take handedness into consideration (9, 24, 25), we still believe our results are worth reporting. However, we should point that most studies had a predominant right-handed population, and the asymmetric cerebral alterations observed might be due to handedness, and not necessarily side of disease onset. Other neurodegenerative diseases, such as Alzheimer’s disease, are not commonly investigated for laterality, and most studies focus on patterns of disease progression, showing that early atrophy occurs on medial temporal areas and advances toward frontal and parietal lobes (26). Our next step is to extend this investigation using a larger sample taking into account dominance and side of disease onset. There should also be a more detailed study of cognitive functions, especially language. Further research is needed to clarify the differential patterns and importance of brain involvement in RDO and LDO in PD.

## Conclusion

Brain atrophy in subjects with PD is bilateral, and regardless of the side of disease onset, atrophy seems more severe in the left hemisphere. It remains unclear if this left-sided predominance is secondary to hand dominance or if it results from a specific characteristic of disease progression.

## Author Contributions

(1) Research project: (A) Conception: AD, MS, CP, FC; (B) Organization: AD, MS, LP, LC, RG, PA, FC; and (C) Execution: MS, CP, LP, LC, RG, PA, BC, AA-F, AD. (2) Statistical analysis: (A) Design: MS, AD, BC, LP, LC, RG, PA, FC; (B) Execution: MS, CP, BC, RG, AD; and (C) Review and critique: AD, BC, RG, FC. (3) Manuscript: (A) Writing of the first draft: MS, AD and (B) Review and critique: AD, FC, LP, CP, LC, RG, PA, BC, AA-F.

## Conflict of Interest Statement

None of the authors have any commercial or financial relationships that could be a conflict of interest related to the development of the manuscript. Financial disclosures: MS, RG, and CP: research grant from FAPESP; LC, PA, and LP: educational grant from Ipsen; BC and AA-F: none; FC: supported by grants from FAPESP and CNPq, Brazil; and AD: research grant from FAPESP and CNPq, Brazil.

## Footnotes

^1^http://people.cas.sc.edu/rorden/mricron/dcm2nii.html

^2^http://www.fil.ion.ucl.ac.uk/spm
